# Supplementary figures and images for: Development of Microalgae Biosensor Chip by Incorporating Microarray Oxygen Sensor for Pesticides Sensing
Source: Biosensors (Basel). 2019 Nov 12;9(4):133. doi: 10.3390/bios9040133 (PMC6956216; doi:10.3390/bios9040133)

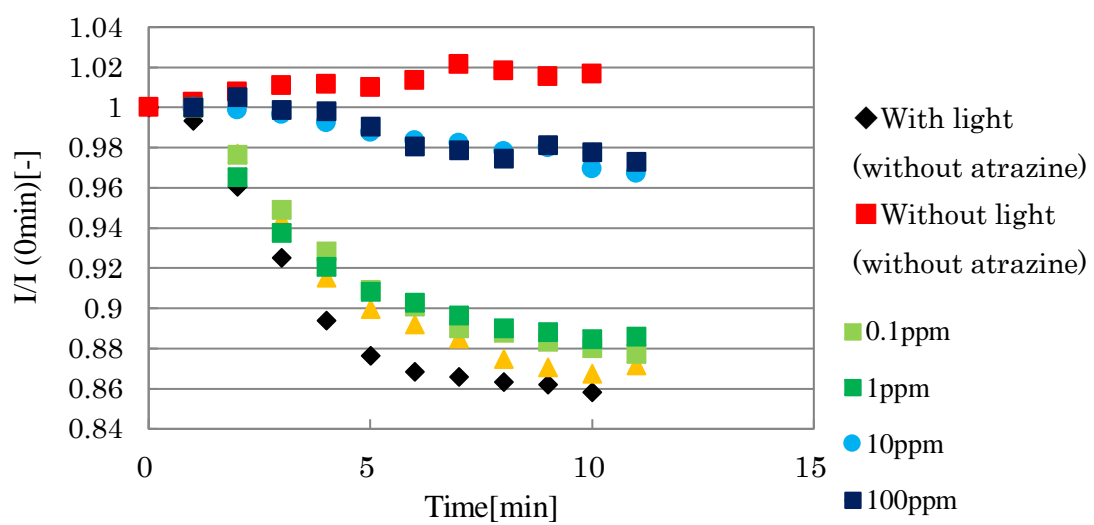

Figure A-1. Biosensor response curves in different atrazine concentration (0–100 ppm).

Supplement: Supplementary file 1 [file biosensors-09-00133-s001.pdf]
